# Induction of Cyclooxygenase-2 Expression by Hepatitis B Virus Depends on Demethylation-associated Recruitment of Transcription Factors to the Promoter

**DOI:** 10.1186/1743-422X-8-118

**Published:** 2011-03-14

**Authors:** Xin Yue, Fang Yang, Yongbo Yang, Yongxin Mu, Wei Sun, Wei Li, Dongping Xu, Jianguo Wu, Ying Zhu

**Affiliations:** 1 State Key Laboratory of Virology and College of Life Sciences, Chinese-French Liver Disease Research Institute of Wuhan University (Zhongnan Hospital), Wuhan University, Wuhan 430072, PR China; 2 Research Group for Viral Hepatitis, Institute of Infectious Diseases, Beijing 302 Hospital, Beijing 100039, PR China

## Abstract

**Background:**

The hepatitis B virus (HBV) is a major etiological factor of inflammation and damage to the liver resulting in hepatocellular carcinoma. Transcription factors play important roles in the disordered gene expression and liver injury caused by HBV. However, the molecular mechanisms behind this observation have not been defined.

**Results:**

In this study, we observed that circulating prostaglandin (PGE) 2 synthesis was increased in patients with chronic hepatitis B infection, and detected elevated cyclooxygenase (COX)-2 expression in HBV- and HBx-expressing liver cells. Likewise, the association of HBx with C/EBPβ contributed to the induction of COX-2. The COX-2 promoter was hypomethylated in HBV-positive cells, and specific demethylation of CpG dinucleotides within each of the two NF-AT sites in the COX-2 promoter resulted in the increased binding affinity of NF-AT to the cognate sites in the promoter, followed by increased COX-2 expression and PGE2 accumulation. The DNA methylatransferase DNMT3B played a key role in the methylation of the COX-2 promoter, and its decreased binding to the promoter was responsible for the regional demethylation of CpG sites, and for the increased binding of transcription factors in HBV-positive cells.

**Conclusion:**

Our results indicate that upregulation of COX-2 by HBV and HBx is mediated by both demethylation events and recruitment of multiple transcription factors binding to the promoter.

## Background

Human hepatitis B virus (HBV) is a non-cytopathic partially double-stranded DNA virus that causes acute and chronic liver disease. HBV infection affects approximately 350 million people in the world [1], and is strongly associated with the development of liver cirrhosis and hepatocelluar carcinoma (HCC) [2]. Even though multiple factors have been implicated in HBV-associated pathogenesis and carcinogenesis, the exact molecular mechanism underlying chronic HBV-induced liver cancer remains largely unknown [3].

Data from HBV-infected individuals and *in vitro *experiments have shown that activation of the intrahepatic cytokine network may play an important role in pathogenesis of acute and chronic liver inflammation and injury caused by HBV infection. Expression of tumor necrosis factor-α [4,5], interleukin-6 [6], inducible nitric oxide synthase [7,8] and cyclooxygenase-2 [8,9] are increased in response to HBV infection, or are involved in HBV-associated HCC development.

Cyclooxygenase-2 (COX-2) is an inducible COX isoform required for the biosynthesis of prostaglandins, such as PGE2, and is primarily involved in pathological processes such as inflammation, fibrogenesis, and carcinogenesis [10]. Increases in COX-2 and PGE2 have been reported in chronic hepatitis, liver cirrhosis, and hepatocellular carcinoma [8,9]. COX-2 is induced at the transcriptional level by a variety of stimuli including cytokines, mitogens, and growth factors [10,11]. Its promoter region contains a TATA box, an E box, and binding sites for a number of transcription factors such as NF-κB, NF-AT/AP1, NF-IL6/C/EBP, and CREB [10-13].

It is well accepted that viral proteins stimulate COX-2 expression, such as latent membrane protein 1 of Epstein-Barr virus (EBV) [14], Gp120 [15] and Tat of human immunodeficiency virus (HIV) [16], and core and NS5A of human hepatitis C virus (HCV) [17]. We have previously demonstrated that NS3 of HCV [18] and the spike and nucleocapsid proteins of SARS-associated coronavirus (SARS-CoV) [19,20] induce COX-2 expression in virus infected cells. Upregulation of COX-2 was also observed in liver tissues chronically infected with HBV [21]. Recent studies have shown that HBx, a 17.5 kDa multifunctional transactivator encoded by the HBV genome [22], is likely the most important determinant of COX-2 induction in human cells infected with HBV. Importantly, this of COX-2 expression may contribute to the pathogenesis of HBV [23].

DNA methylation and histone modifications represent the major epigenetic mechanisms implicated in the regulation of gene transcription in mammals. DNA methylation is a predominant mechanism employed to inactivate relevant genes during tumorigenesis associated with HCC. DNA methylation occurs at carbon 5 of cytosine, primarily in the context of the CpG dinucleotide. Recent findings support the hypothesis that hypomethylation of the DNA surrounding the proximal promoter region is a prerequisite for gene activation, whereas hypermethylation leads to gene silencing [24]. Mechanistically, there are a number of ways in which DNA methylation can repress transcription. For example, many of the *trans *factors known to bind to sequences containing CpG dinucleotides do not bind when the CpG doublets are methylated [25]. Identification of methylated regions in the COX-2 promoter during HBV infection will aid in understanding the mechanisms by which this gene is regulated.

To study the influence of HBV on the expression of COX-2, we first examined the amount of PGE2 in the peripheral blood serum of patients with chronic hepatitis B. We also analyzed the mechanism of COX-2 upregulation in HBV-positive cells as well as in HBx-transfected cells. Our results showed that COX-2 expression was induced in both HBV-positive and HBx-expressing cells, and that activation of the COX-2 promoter by HBx depended on C/EBP and CREB binding sites, in which C/EBPβ played an important role. HBx directly interacted with C/EBPβ, and enhanced its binding to the COX-2 promoter. DNA methylation analysis revealed that global hypomethylation of the COX-2 promoter and specific demethylation of the NF-AT binding sites occurred in HBV-positive cells. Among the three DNA methyltransferases (DNMTs) present in mammalian cells, DNMT3B played a key role in COX-2 promoter methylation, and its decreased affinity for the promoter was responsible for the specific regional demethylation and the increased binding of transcription factors. Together, this study shows that both aberrant epigenetic modifications and transcription factors contribute to the HBV-induced COX-2 expression.

## Results

### Elevated serum PGE2 in HBV patients

High level expression of circulating PGE2 was observed in patients with chronic HBV infection. As shown in Table 1 the serum levels of PGE2 were significantly higher in patients with chronic hepatitis B infection in comparison with healthy control individuals (mean ± SEM 1393.9 ± 834.5 vs. 161.6 ± 79.2 pg/mL P < 0.01), suggesting that HBV infection resulted in upregulation of COX-2.

**Table 1:** Demographic and baseline characteristics, serum levels of PGE2 from HBV patients and healthy individuals

Characteristic	Healthy individuals (N = 102)	Patients (N = 107)
Age (years)	42.5 ± 12.7	45.4 ± 13.8

Gender (male/female)	83/19	89/18

Race or ethnic group-no. (%) Asian	102 (100)	107 (100)

HBeAg (+/-)	0/102	42/65

HBV genotype (b/c)	NA	16/91

WBC (cells/μl)	3615 ± 860	7602 ± 7640

ALT (U/L)	< 30	156.9 ± 174.9

HBV DNA (copies/ml)	< 500	9.9E + 07 ± 4.7E + 08

PGE2 (pg/ml)	161.6 ± 79.2	1393.9 ± 834.5

### HBV and HBx expression upregulates COX-2 expression and COX-2 promoter activity

**Figure 1:**
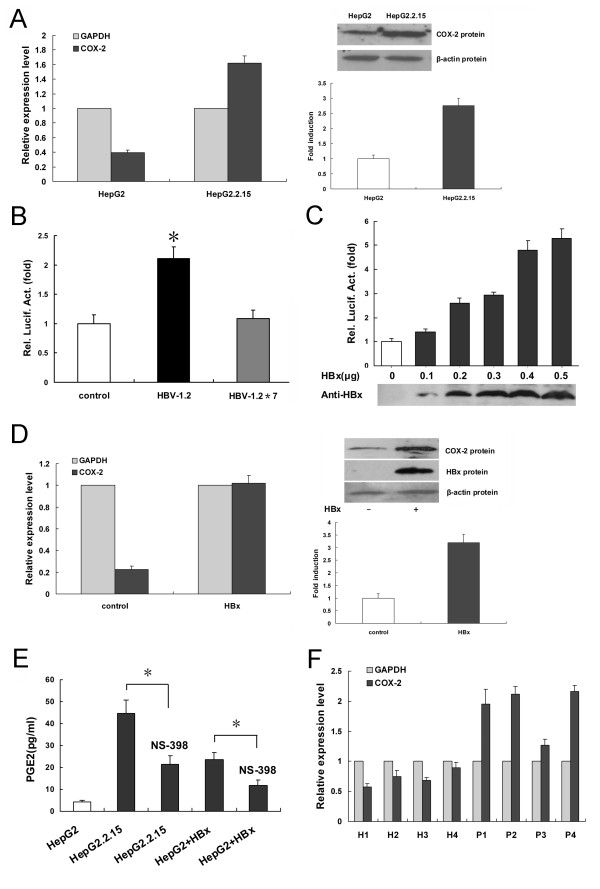
**COX-2 is upregulated in HBV-positive cells**. (A) HepG2 or HepG2.2.15 cells were serum-starved for 24 hours, after which COX-2 mRNA and protein were detected by qRT-PCR and western blot respectively. (B) COX-2 promoter activity was measured by transfecting HepG2 cells with pCOX-2-Luc and pHBV-1.2 or pHBV-1.2*7, an HBx-deficient HBV mutant. A *Renilla *luciferase reporter vector pRL-TK was used as internal control. Luciferase activity in each sample was measured at 48 hours post-transfection. All data were normalized to the *Renilla *luciferase value, and are expressed as mean ± SD, n = 3 (*P < 0.05). (C) HepG2 cells were cotransfected with different amounts of pCMV-HBx, and with pCOX-2-Luc and pRL-TK. Relative luciferase activities were measured at 48 hours post-transfection. Results are presented as fold induction over values obtained in cells not transfected with HBx, and as mean ± SD, n = 3. (D) HepG2 cells were transfected with pCMV-HBx. 48 hours after transfection COX-2 mRNA and protein were determined by qRT-PCR and western blot respectively. (E) PGE2 levels were detected in HepG2.2.15 or HepG2 cells transfected with pCMV-HBx compared with mock transfected cells. Cells were treated with the COX-2 inhibitor NS-398 at 100 μM for 24 hours where indicated. Results are presented as mean ± SD, n = 3 (*P < 0.05). (F) The PBMC lysates from healthy individuals (H1 to H4) or HBV patients (P1 to P4) were used to extract total RNA, COX-2 mRNA was then detected by qRT-PCR.

Simultaneous expression of HBx and COX-2 has been observed in liver specimens from chronic HBV patients [26]. To test if expression of HBV influences COX-2, expression of COX-2 in HepG2 human hepatoma cells was compared with that in HepG2.2.15 cells, which contain an integrated HBV genome. We analyzed COX-2 mRNA expression by quantitative RT-PCR, and detected an increase in COX-2 mRNA in HepG2.2.15 cells as compared to HepG2 cells, which do not express HBV (Figure 1A). The amount of COX-2 protein was next estimated by Western blot analysis, and endogenous COX-2 protein was increased in HepG2.2.15 cells (Figure 1A), suggesting that the presence of HBV regulates COX-2 expression. To test if the upregulation of COX-2 correlates with the expression of HBx, a reporter plasmid in which the COX-2 promoter was cloned upstream of the luciferase gene (pCOX-2-Luc) was transfected with pHBV-1.2 or pHBV-1.2*7 and the control plasmid pRL-TK into HepG2 human hepatoma cells, which support HBV replication. pHBV-1.2 contains 1.2-fold length of the HBV genome and retains the ability to produce mature HBV virions. pHBV-1.2*7 is an HBV mutant that does not express HBx. Luciferase activity was measured in each sample, and results showed that wild-type HBV activated COX-2 promoter whereas the HBV mutant failed to induce COX-2 promoter activation (Figure 1B). Since expression of luciferase in these cells could only come from activity at the COX-2 promoter in the reporter plasmid, it is likely that COX-2 expression is regulated by HBx at the level of transcription. We next transfected HepG2 cells with pCOX-2-Luc and increasing concentrations of the pCMV-HBx plasmid, and found that HBx stimulated the COX-2 promoter in a dose-dependent manner (Figure 1C). To further confirm the effects of HBx on COX-2 expression, the pCMV-HBx plasmid was transiently transfected into HepG2 cells. qRT-PCR and western blot analysis showed elevated level of COX-2 mRNA and protein in HBx transfeted cells (Figure 1D).

COX-2 is a key enzyme involved in prostaglandin synthesis, as it converts arachidonic acid to PGE2 and other prostanoids involved in inflammation and tumor development [10,27]. Elevated COX-2 activity in HBV or HBx expressing cells was next monitored by measuring the accumulation of secreted PGE2 in culture supernatants from HepG2.2.15 cells, or from HepG2 cells transfected with pCMV-HBx. As shown in Figure 1E, the concentration of PGE2 was higher in HBV-positive or HBx-expressing cells as opposed to control cells. We next confirmed the specificity of COX-2 regulation for PGE2 secretion by treating the cells with NS-398, a selective inhibitor of COX-2. We found that treatment with NS-398 significantly reduced the amount of secreted PGE2 as compared to untreated cells, confirming the specific relationship between HBV- and HBx-induced COX-2 expression and PGE2 secretion (Figure 1E).

To show the consistency of the above results with clinical manifestations of hepatitis B, we performed qRT-PCR analysis of peripheral blood mononuclear cells (PBMC) from hepatitis B patients. Results showed elevated levels of COX-2 mRNA in PBMC of these people compared with healthy individuals (Figure 1F).

### C/EBPβ plays a role in HBx-induced COX-2 promoter activation

**Figure 2:**
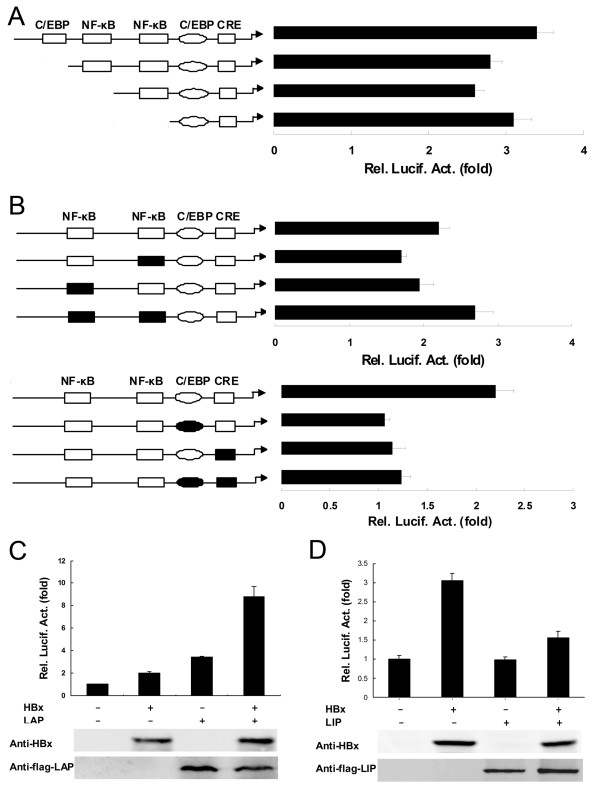
**C/EBPβ plays a role in HBx-induced COX-2 promoter activation**. (A) HepG2 cells were transfected with luciferase reporter plasmids containing the wild-type COX-2 promoter (-891/+9), or with plasmids containing deletion mutants of the COX-2 promoter, along with pCMV-HBx and pRL-TK. Luciferase activites were measured in each sample after serum starvation for 24 hours, and were compared to those obtained from control cells transfected with empty vector. All data were normalized to the Renilla luciferase value and are presented as mean ± SD, n = 3. (B) HepG2 cells were transfected with luciferase reporter plasmids containing the wild-type COX-2 promoter, or with those containing point mutations in the NFκB, C/EBP and CRE binding sties in the COX-2 promoter, along with pCMV-HBx and pRL-TK. Luciferase activities were measured as described in (A) (*P < 0.05). (C) HepG2 cells were transfected with pCOX-2-Luc, pCMV-HBx, pCMV-LAP, and pRL-TK, and luciferase activity was measured as described in (A) (*P < 0.05). (D) HepG2 cells were transfected with pCOX-2-Luc, pCMV-HBx, pCMV-LIP and pRL-TK, and luciferase activity was measured as described in (A) (*P < 0.05).

C/EBP, NF-κB, and CRE are important regulators of the COX-2 promoter [10-13]. To identify the *cis*-regulatory elements in the COX-2 promoter that are responsive to HBx, mutant reporter plasmids were constructed in which the binding site of these factors was altered in the COX-2 promoter. These altered promoters were cloned upstream of the luciferase gene, and were transfected into HepG2 cells with pCMV-HBx. Deletion or mutation of the NF-κB binding sites had little effect on the activation of the COX-2 promoter by HBx (Figure 2A and 2B), which is consistent with prior studies [23]. A single mutation in the C/EBP, CRE, or double mutation in both, nearly abolished the activation of the COX-2 promoter by HBx (Figure 2B). These results show that the C/EBP and CRE binding sites are required for COX-2 promoter activation by HBx, suggesting that HBx induces COX-2 expression in a C/EBP- and CRE-dependent manner.

The C/EBP transcription factor family contains at least six members (C/EBPα-C/EBPζ). C/EBPβ is widely involved in inflammation and regulates gene transcription in response to a variety of stimuli [28,29]. We next investigated the role of C/EBPβ in the activation of COX-2 by HBx. At least three isoforms of C/EBPβ, LAP* (38 kDa), LAP (35 kDa), and LIP (20 kDa) are constitutively expressed in a variety of cell types. LAP and LAP* both contain a transactivation domain and a DNA binding domain, whereas LIP only contains a DNA binding domain [28-30]. HepG2 cells were cotransfected with pCOX-2-Luc, pCMV-HBx, and pCMV-LAP or pCMV-LIP. Luciferase activity in each sample was measured after 24 hours, and the expression of each cognate protein was confirmed by Western blot. A synergistic effect between HBx and LAP on the COX-2 promoter was observed (Figure 2C). As expected, LIP inhibited HBx-induced COX-2 promoter activation (Figure 2D), likely due to the absence of a DNA-binding domain. These data indicate that HBx and C/EBPβ cooperatively activate COX-2 transcription.

### HBx directly interacts with C/EBPβ and stimulates binding of C/EBPβ to the COX-2 promoter

**Figure 3:**
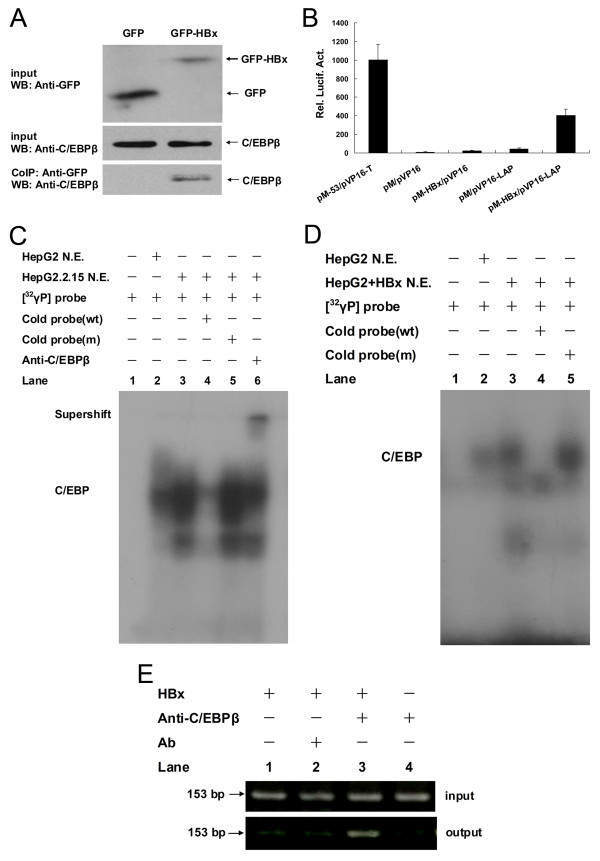
**HBx directly interacts with C/EBPβ and stimulates binding of C/EBPβ to the COX-2 promoter**. (A) HepG2 cells were transfected with plasmids encoding GFP-HBx or GFP alone. Cellular extracts were prepared and co-immunoprecipitated with anti-GFP antibody, and proteins were resolved by SDS-PAGE. Blots were incubated with the antibodies indicated in the figure, and were developed with chemiluminescence. (B) HepG2 cells were transfected with pG5-Luc, pRL-TK, pVP16-LAP, pM-HBx, or empty vectors, as indicated. Luciferase activity was measured in each sample, and was compared to those obtained from control cells. All data are normalized to the *Renilla *luciferase value, and are presented as mean ± SD, n = 3 (*P < 0.05). (C) Nuclear extracts were prepared from HepG2.2.15 and HepG2 cells, and were mixed with a ^32^P-labeled C/EBP probe and analyzed by electrophoretic mobility shift assay (EMSA). Excess non-labeled C/EBP probe (100-fold) was used as a competitor. Polyclonal anti-C/EBPβ was incubated with nuclear extracts prior to addition of the probes, as indicated. (D) Nuclear extracts were prepared from HepG2 cells transfected with pCMV-HBx, and with mock transfected cells, and were mixed with a ^32^P-labeled C/EBP probe and analyzed by EMSA. Excess non-labeled C/EBP probe (100-fold) was used as a competitor. (E) HepG2 cells were transfected for 48 hours with pCMV-HBx or pCMV-Tag2A, and ChIP assays were performed using 2 μg of anti-C/EBPβ. Normal rabbit IgG was used as control. Immunoprecipitated DNA or control DNA was collected and amplified using specific primers for the C/EBP binding site in the COX-2 promoter (nucleotides -155 to -2).

As a transcription coactivator, HBx interacts with several members of the basal transcriptional apparatus. We next tested if HBx directly interacts with C/EBPβ, as it is a component of this apparatus. HepG2 cells were transfected with plasmids encoding GFP-HBx or GFP alone, and cell lysates were immunoprecipitated with anti-GFP antibodies followed by Western blotting with antibody specific for C/EBPβ. Our results showed that HBx co-immunoprecipitated with endogenous C/EBPβ (Figure 3A), confirming an interaction between HBx and the basal transcriptional machinery.

To further confirm an interaction between HBx and C/EBPβ, we employed the mammalian two-hybrid assay in HepG2 cells. We constructed a vector containing the activation domain of VP16 fused with the N terminus of LAP (pVP16-LAP), and a vector containing HBx fused at its N terminus to the DNA-binding domain of Gal4 (pM-HBx). In this assay, if an interaction occurs between HBx and LAP, the activation domain of VP16 will be brought into contact with its DNA binding domain, thus inducing transcription of the luciferase gene in the pG5-Luc reporter plasmid. HepG2 cells were transfected with these plasmids, in addition to the control plasmid pRL-TK, and our results showed that luciferase expression in cells transfected with both pM-HBx and pVP16-LAP was much higher than in control cells (Figure 3B), confirming a direct interaction between HBx and C/EBPβ.

Prior studies have suggested that HBx may interact with bZip factors through the bZip domain, which promotes the binding to and activation of target promoters [31]. We therefore used EMSA analyses to investigate the possibility that C/EBP factors activated by HBx bind DNA. Nuclear extracts were prepared from HepG2 and HepG2.2.15 cells (Figure 3C), or from HepG2 cells transfected with pCMV-HBx (Figure 3D), and were incubated with a ^32^P-labeled probe representing the C/EBP binding site from the COX-2 promoter. Binding of nuclear factors to the probe was significantly increased in HepG2.2.15 and in HBx-expressing cells (Figure 3C, lanes 2 and 3, and Figure 3D, lanes 2 and 3). This interaction was eliminated when unlabeled probe was included in the sample (Figure 3C and 3D, lane 4), but was not affected by the addition of a C/EBP mutant probe (Figure 3C and 3D, lane 5). Supershifted complexes were observed when C/EBPβ antibody was added (Figure 3C, lane 6), confirming that HBx induces the binding of C/EBPβ to the COX-2 promoter.

We next confirmed the HBx-induced C/EBPβ binding to the COX-2 promoter using chromatin immunoprecipitation assays (ChIP). Chromatin fragments were prepared from HepG2 cells transfected with pCMV-HBx, and were immunoprecipitated with C/EBPβ antibody. The COX-2 promoter region, consisting of nucleotides -155 to -2 relative to the transcription start site and containing C/EBP binding sequences, was amplified from the chromatin precipitates by PCR. We observed that the amount of C/EBPβ bound to the COX-2 promoter was higher in the presence of HBx (Figure 3E, lane 3) as opposed to cells not expressing HBx (Figure 3E, lane 4). Taken together, these data suggest that HBx stimulates the binding of C/EBPβ to the COX-2 promoter.

### Demethylation activates COX-2 promoter activity in HBV-positive cells

**Figure 4:**
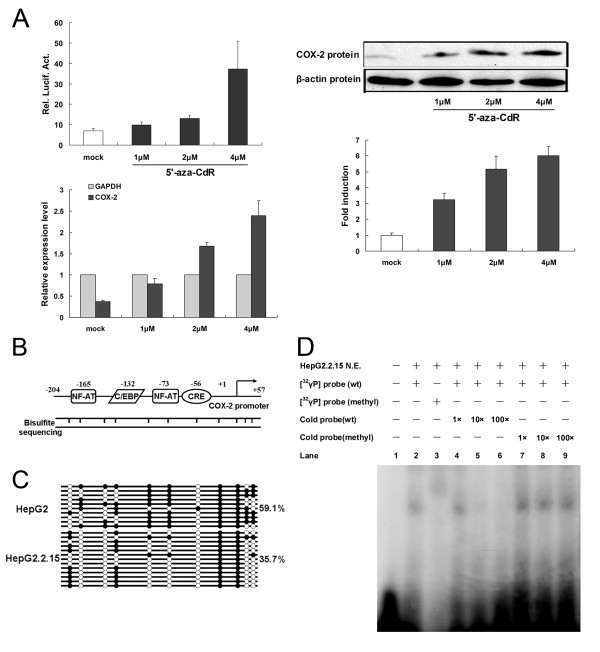
**Demethylation activates COX-2 promoter activity in HBV-positive cells**. (A) Cells were seeded at a density of 1 × 10^6 ^cells/10 cm dish and transfected with pCOX-2-Luc and pRL-TK. 5-aza-CdR was added to a final concentration of 1, 2, or 4 μM at 6 hours post-transfection. Cells were allowed to grow for another 24 hours for qRT-PCR, and 48 hours for luciferase assays and Western blots. Luciferase data were normalized to that of the *Renilla *luciferase value, and are presented as mean ± SD, n = 3. (B) Genomic sequencing data of the COX-2 promoter in HepG2 cells. Potential methylation sites are indicated with vertical lines. (C) Genomic DNA from HepG2 and HepG2.2.15 cells was treated with sodium bisulfite and PCR amplified for the COX-2 promoter. Amplicons were cloned and sequenced, and each row of circles represents a single sequenced clone. The solid circles indicate methylated cytosines, while the open circles indicate unmethylated cytosines. (D) Nuclear extracts were prepared from HepG2.2.15 cells and were mixed with a ^32^P-labeled unmethylated NF-AT probe, or with an *in vitro *methylated NF-AT probe, and analyzed by EMSA. Excess non-labeled NF-AT probe was used as a competitor. wt: wild-type; methyl: methylated.

HBV infection and expression of HBx cause aberrant methylation of tumor gene promoters [32], and the demethylating agent 5-aza-CdR has been previously used to investigate the role of methylation on gene expression [33,34]. Here, HepG2 cells were transfected with pCOX-2-Luc and pRL-TK, and 5-aza-CdR was added to a final concentration of 1, 2, or 4 μM after 6 hours. At 24 and 48 hours post-transfection, cells were harvested for qRT-PCR, or Western blot and luciferase assays, respectively. As shown in Figure 4A, the addition of 5-aza-CdR stimulated both promoter activity and the expression level of COX-2 in a dose-dependent manner.

We next used bisulfate sequencing to obtain more precise information concerning the methylation status of the COX-2 promoter, as this method permits high sensitivity mapping of methylated cytosine residues [35]. As shown in Figure 4B, the COX-2 promoter was sequenced from genomic DNA isolated from HepG2 cells, from nucleotides -204 to +57, and was found to contain 11 methylatable CpG sites. Bisulfite-modified genomic DNA was then PCR amplified for the COX-2 promoter, and the products were cloned and sequenced. The percentage of methylated CpGs was significantly lower in HepG2.2.15 cells (35.7%) than in HepG2 cells (59.1%) (Figure 4C). Importantly, two known NF-AT motifs (-73 and -165), both of which contain a CpG site, were demethylated on the COX-2 promoter in HBV-expressing HepG2.2.15 cells, indicating that site-specific demethylation of NF-AT sites are essential for NF-AT binding and COX-2 promoter activation.

We next tested the influence of methylation on the affinity of NF-AT to its binding motif on the COX-2 promoter. We performed EMSA using nuclear extracts from HepG2.2.15 cells and a NF-AT probe spanning nucleotides -85 to -66 of the COX-2 promoter. As shown in Figure 4D, methylation of these nucleotides inhibited the formation of DNA-protein complexes, suggesting that the binding affinity between NF-AT and the COX-2 promoter after demethylation of the CpG dinucleotide on its binding motif was enhanced. Taken together, these results suggest that hypomethylation of the COX-2 promoter, especially at the NF-AT binding sites, activates COX-2 promoter activity in HBV-expressing cells.

### DNMT3B plays a role in COX-2 promoter methylation

**Figure 5:**
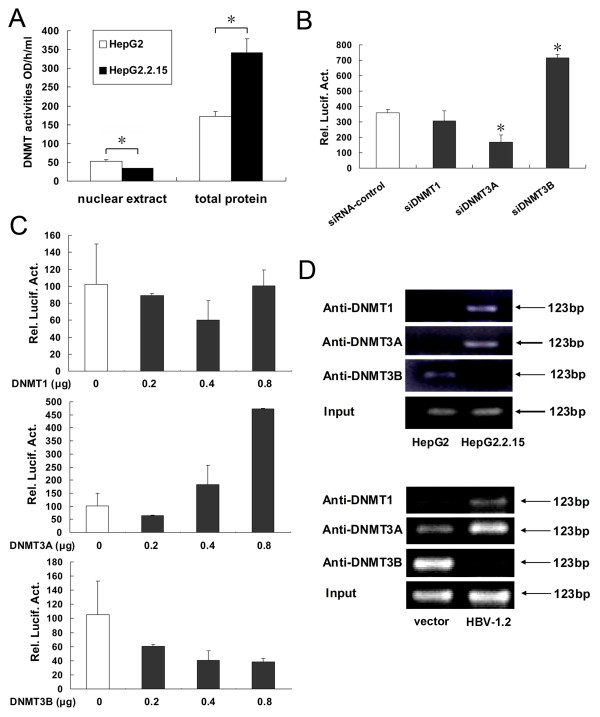
**DNMT3B is responsible for the hypomethylation of COX-2 promoter**. (A) DNMT activities of both total cellular and nuclear extracts from HepG2 and HepG2.2.15 cells were measured using the Epiquik DNA Methyltransferase activity assay kit (*P < 0.05). (B) HepG2 cells were transfected with pCOX-2-Luc, pRL-TK, siDNMT1, siDNMT3A or siDNMT3B. Luciferase activites were measured after serum starvation for 24 hours, and all data were normalized to the *Renilla *luciferase value, and are presented as mean ± SD, n = 3 (*P < 0.05). (C) HepG2 cells were transfected with pCOX-2-Luc, pCDNA-DNMT1, pCDNA-DNMT3A or pCDNA-DNMT3B. Luciferase activites were measured as in (B). (D) HepG2 or HepG2.2.15 cells and HepG2 cells transfected with pHBV-1.2 or control vector were used in ChIP assays using 2 μg anti-DNMT1, -DNMT3A, or -DNMT3B. Immunoprecipitated DNA or control DNA was collected and amplified using specific primers for the COX-2 promoter (nucleotides -177 to -56).

In mammalian cells, precise somatic DNA methylation patterns are established during gametogenesis and early embryogenesis by three known DNA methytransferases (DNMT), specifically DNMT1, DNMT3A, DNMT3B [36]. All of these DNMTs are overexpressed in cancers [37]. To test if HBV initiates transcription by demethylation, we examined potential differences in DNMT activity in HepG2 and HepG2.2.1.5 cells (Figure 5A). We found that the activity of total cellular DNMTs were increased in HepG2.2.1.5 cells, but was decreased in the nucleus, suggesting that HBV expression attenuates the activity of nuclear DNMTs which are involved in gene regulation directly.

To determine which DNMT is involved in activating the COX-2 promoter, HepG2 cells were transfected with pCOX-2-Luc alone or with siRNA specific for DNMT1, DNMT3A, or DNMT3B. As shown in Figure 5B, COX-2 promoter activity was downregulated after knock-down of DNMT3A, and was upregulated by knock-down of DNMT3B. Co-transfection of DNMT expression plasmids with pCOX-2-Luc revealed that DNMT3B decreased COX-2 promoter activity in a dose-dependent manner, whereas DNMT3A increased in activity (Figure 5C). We next investigated the recruitment of these three DNMTs to the COX-2 promoter by ChIP assay. Chromatin fragments were prepared from HepG2 and HepG2.2.15 cells and were immunoprecipitated with antibodies specific for DNMT1, DNMT3A, or DNMT3B. The COX-2 promoter was then amplified by PCR, and we found that DNMT3B binding to the COX-2 promoter was reduced in HBV-expressing HepG2.2.15 cells, whereas that of DNMT3A and DNMT1 was increased (Figure 5D). Same results were shown in pHBV-1.2 transfected HepG2 cells (Figure 5D). A prior study has shown diminished expression of DNMT3B and increased expression of DNMT1 and 3A in HBx-expressing cells [38]. So the different DNA binding affinities may due to different expressions of DNMTs. These findings, combined with previous results that the COX-2 promoter is demethylated in HBV-expressing cells, suggest that DNMT3B but not DNMT1 or DNMT3A plays a key role in COX-2 promoter methylation, and that the decreased activity of DNMT3B in the nucleus is likely responsible for hypomethylation of the COX-2 promoter.

## Discussion

In this study, we observed significant increases in PEG2 synthesis in patients with chronic hepatitis B infection, and also detected elevated COX-2 expression in HBV and HBx-expressing liver cells. We demonstrated a previously unrecognized mechanism of COX-2 regulation, where demethylation-linked recruitment of multiple transcription factors to the COX-2 promoter activated HBV-induced COX-2 expression. Demethylation of CpG dinucleotides within the NF-AT binding sites in the COX-2 promoter resulted in the increased binding affinity of NF-AT to the cognate sites in the promoter, followed by increased COX-2 expression and PGE2 accumulation. The association of HBx with C/EBPβ also contributed to the induction of COX-2. Likewise, DNMT3B played a key role in the methylation of the COX-2 promoter, as the reduced binding between DNMT3B and the COX-2 promoter was responsible for the regional demethylation of the CpG sites, and for the increased binding of transcription factors in HBV-positive cells.

COX-2, the inducible isoform of COX, is highly expressed in response to various pro-inflammatory stimuli and other cellular stresses [10,11], and previous studies have shown increased levels of COX-2 in a variety of human cancers [27]. Importantly, overexpression of COX-2 is closely linked to viral infection [14-17]. Chronic infection with HBV or HCV is the leading cause of liver cancer [1-3], and elevated expression of COX-2 has been described in different liver cirrhosis animal models and liver tissue from cancer patients with chronic HBV or HCV infections [8,9,17,21]. HBx is a transcriptional activator that is required for viral infection and contributes to HBV-associated hepatocarcinogenesis [39]. Accumulating evidence suggests that HBx activates a large number of cellular genes related to HBV-associated HCC, including the COX-2 gene [22,40]. Coexpression of HBx and COX-2 was detected in chronic liver diseases associated with HBV infection [26], and HBx also promotes tumor cell invasion by inducing membrane type 1 matrix metalloproteinase and COX-2 [23]. However, the molecular details of COX-2 activation during chronic HBV infection remain to be defined.

It has been well established that NF-κB, C/EBP, and CREB are the primary transcription factors responsible for the regulation of COX-2 expression [10-13]. In this study we demonstrated that the C/EBP and CRE binding sites, but not those of NF-κB, were critical for HBx-induced COX-2 promoter activation. It is possible that NF-κB might be involved in COX-2 expression, but that it is not directly responsible for that induced by HBx.

We investigated the effect of HBx and the C/EBPβ isoform on COX-2 promoter activity, as C/EBPβ is widely involved in inflammation and cellular proliferation of a number of cell types, including hepatocytes, and regulates gene transcription in response to a varity of stimuli [28,29]. We found that the effects of different isoforms of C/EBPβ played diverse roles in this process. LAP (transcription activator) and HBx had a synergistic effect on COX-2 promoter activation, whereas LIP (transcription repressor) led to a decrease in HBx-induced COX-2 expression. These data suggest that C/EBPβ, cooperatively with HBx, activates COX-2 transcription through the transactivation domain of C/EBPβ. Likewise, bZip transcription factors, such as CREB and C/EBP, were reported to interact with HBx and target a variety of promoters [31,40,41]. We detected a direct interaction between HBx and C/EBPβ, which may contribute to the reinforcement of C/EBPβ binding to the COX-2 promoter, and the subsequent upregulation of COX-2 expression.

Others have confirmed a direct correlation between the expression of a given gene and its methylation status [42], and previous studies have proposed promoter methylation as a mechanism to regulate COX-2 expression in tumors [43-45]. Likewise, other studies have shown that DNA methylation of certain promoters is associated with HBV infection [32]. Since many of the *trans *factors known to bind to sequences containing CpG dinucleotides do not bind when such regions are methylated, we looked for relationships between transcription factor binding and aberrant epigenetic modifications in the COX-2 promoter during HBV infection. Bisulfite sequencing analysis of the CpG island in the promoter (nucleotides -204 to +57) revealed the hypomethylation of the COX-2 promoter in HBV-expressing cells. More importantly, demethylation of two NF-AT motifs, which contained CpG sites, was detected in these cells, and EMSA data indicated a higher affinity of NF-AT for the demethylated motif of the COX-2 promoter. As a previous report found that HBx activates the COX-2 promoter in an NF-AT dependent manner [23], our results describe a possible mechanism for the role of NF-AT in this process. Considering the complex interactions of HBx, C/EBPβ and NF-AT, demethylation of NF-AT sites might lead to the binding of different *trans *factors. These results suggest a potential mechanism for the C/EBPβ- and NF-AT-dependent COX-2 activation by HBV and HBx.

DNMT1 is activated by the Ras-AP-1 signaling pathway [46], Hypermethylation of tumor suppressor genes are mediated by DNMT3A or DNMT3B, and diminished expression of DNMT3B was detected in HBx-expressing liver cell lines, resulting in genomic hypomethylation [38]. We investigated the role of these three DNMTs in the epigenetic modifications of the COX-2 promoter. We found that DNMT3B but not DNMT1 or DNMT3A was critical for methylation of the COX-2 promoter, and ChIP assays showed that the binding of DNMT3B to the COX-2 promoter was decreased in HBV-expressing cells. A recent study demonstrated the interaction of HBx with DNMT3A and unraveled a possible mechanism involved in HBx-mediated epigenetic modifications and gene regulation [47]. In our study, we investigated the role of three DNMTs (DNMT1, DNMT3A, DNM3B) in the epigenetic modifications of the COX-2 promoter through overexpression and siRNA technology. Considering both the binding of DNMT3B and the total DNMTs activities in the nucleus of HBV-expressing cells were decreased, our results indicate that DNMT3B but not DNMT1 or DNMT3A plays a major role in COX-2 promoter methylation. The increased binding of DNMT1 and DNMT3A to COX-2 promoter in HBV-expressing cells indicates the complex situation in HBV-mediated epigenetic modifications and gene regulation. It is possible that DNMT3A may play a minor role in controlling COX-2 expression through another mechanism. Other researches have showed the importance of mitochondrial pathway in HBx-induced COX-2 expression [48]. We are more concerned about the aberrant epigenetic modifications induced by HBx and its role as a transcriptional coactivator in HBV induced COX-2 expression.

## Conclusions

**Figure 6:**
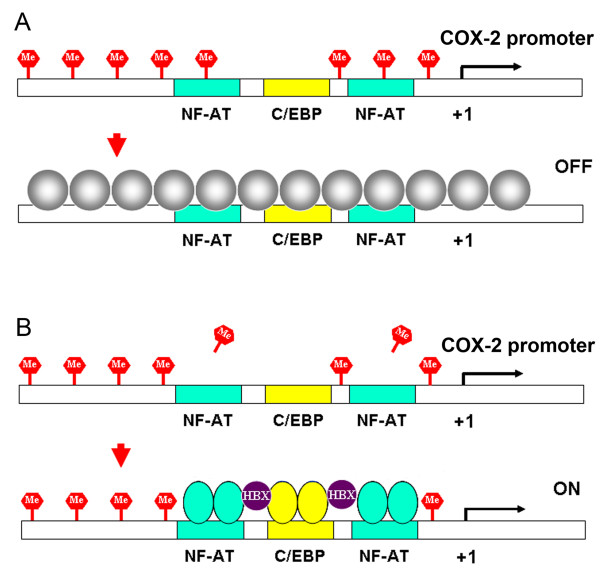
**COX-2 expression induced by demethylation-linked recruitment of multiple transcription factors during HBV infection**. (A) In normal cells, the promoter region of COX-2 is hypermethylated, and transcription repressors such as DNMT3B bind to the methylated CpG sites and repress activity of the COX-2 promoter. (B) After HBV infection, the expression of viral proteins triggers demethylation of the COX-2 promoter by down-regulating DNMT3B. Transcriptional repression is relieved and interactions between HBx and C/EBPβ, NF-AT, and CBP/p300 enhance the recruitment of the complex to the promoter, resulting in expression of the COX-2 gene.

All the data above are consistent with the finding that the COX-2 promoter is hypomethylated in HBV-expressing cells, and suggests a possible mechanism for transcriptional activation of COX-2 by HBV (Figure 6). In normal cells, the COX-2 promoter is hypermethylated and transcription repressors, such as DNMT3B, bind to the methylated CpG sites and repress its activity. After HBV infection, the expression of the virus and HBx trigger demethylation of the COX-2 promoter by down-regulating DNMT3B, which is responsible for the methylation of the promoter. Methylation relieves repression of the promoter and transcription factors such as NF-AT and C/EBPβ are able to bind. Interactions between HBx and C/EBPβ, NF-AT enhance the recruitment of the complex to the COX-2 promoter, the transcription machinery is assembled, and the COX-2 gene is expressed. Such a mechanism provides deeper insights into the molecular mechanism of HBV-induced COX-2 expression.

## Materials and methods

### Clinical samples

Peripheral blood samples were obtained from 107 patients with chronic hepatitis B admitted in Beijing 302 Hospital (89 males and 18 females with mean age 45.4 ± 13.8 years). Matched for sex and age, 102 healthy individuals (83 males and 19 females with mean age 42.5 ± 12.7 years) with no history of liver disease were randomly selected as controls from the local blood donation center. The collection of blood samples for research was approved by the Institutional Review Board of the College of Life Sciences, Wuhan University in accordance with guidelines for the protection of human subjects. Written informed consent was obtained from each participant.

### Plasmids and reagents

A plasmid carrying 1.2-fold length of HBV genome (pHBV-1.2; subtype ayw) and the same plasmid with a stop codon for amino acid 7 of HBx (pHBV-1.2*7) were obtained from Dr. Robert Schneider [49]. Construction of the luciferase reporter vector (pGL3) containing a COX-2 promoter region (nucleotides -891 to +9), its truncated or site-specific mutants, Flag-tagged Human C/EBPβ LAP, and C/EBPβ LIP were reported previously [50]. The HBx gene was amplified by PCR from HepG2.2.15 cells, which contain the HBV genome. The HBx amplicon was cloned into pCMV-Tag2A using *EcoR*I and *Xho*I and into eGFP-C1 using *Xho*I and *Xba*I. HBx and C/EBPβ-LAP were cloned into the mammalian two-hybrid assay plasmids pM (GAL4 DNA-binding domain) and/or pVP16 (VP16 activation domain) (Promega) between the *EcoR*I and *Xba*I sites. DNMT1, DNMT3A, and DNMT3B siRNA plasmids were constructed by ligating the corresponding pairs of oligonucleotides to p*Silencer*™2.1-U6 neo (Ambion, Inc., Austin, TX, USA) based on the target sequences described previously [51].

EGTA, DTT, 5-aza-CdR, NS-398, and antibody against Flag were purchased from Sigma (St. Louis, MO). Antibody against COX-2 was from Cayman (Ann Arbor, MI, USA). Antibodies specific for β-actin, C/EBPβ, GFP, and DNMT1 were purchased from Santa Cruz (Santa Cruz, CA, USA). Antibodies against DNMT3A and DNMT3B were purchased from Cell Signaling Technology (Beverly, MA, USA).

### Cell culture

The human hepatoma cell-line HepG2 was grown in Dulbecco's modified Eagle's medium (DMEM) supplemented with 10% heat-inactivated fetal bovine serum (FBS), 100 U/mL penicillin, and 100 μg/mL streptomycin sulfate at 37°C in 5% CO_2_. The HepG2.2.15 cell-line was derived from HepG2 cells and stably expresses HBV (*ayw*), and was maintained in DMEM containing 400 μg/mL G418.

### Transfection and luciferase reporter gene assays

Cells were plated at density of 4 × 10^5 ^cells per 24-well or 6-well plate, depending on the experiment, and were grown to 80% confluence prior to transfection. Cells were transfected using Lipofectamine 2000 (Invitrogen) for 24 hours, after which they were serum-starved for an additional 24 hours prior to harvest. A *Renilla *luciferase reporter vector pRL-TK was used as internal control. Luciferase activity was measured in each sample 48 hours after transfection using the dual-luciferase reporter assay system (Promega), and *Renilla *luciferase activities were determined as internal controls for transfection efficiency. Assays were performed in triplicate and expressed as mean ± SD relative to the vector or mock control samples, which were set at 100%.

### Quantitative RT-PCR analysis

Quantitative RT-PCR analysis was performed to determine relative mRNA levels. Total RNA was isolated using TRIzol (Invitrogen). Cellular RNA samples were reverse-transcribed using random primers. Real-time PCR was performed in LightCycler 480 (Roche). GAPDH was amplified as an internal control. The primers used were COX-2 up: 5'-TACAATGCTGACTATGGCTAC-3' and COX-2 down: 5'-ACTGATGCGTGAAGTGCTG-3'; GAPDH up: 5'-AAGGCTGTGGGCAAGG-3' and GAPDH down: 5'-TGGAGGAGTGGGTGTCG-3'.

### Western blot analysis

Whole-cell lysates were prepared by lysing cells with PBS pH 7.4 containing 0.01% Triton X-100, 0.01% EDTA, and 10% protease inhibitor cocktail (Roche). Protein concentration in each sample was determined using the Bradford assay kit (Bio-Rad). One hundred micrograms of each sample was subjected to 12% SDS-PAGE, followed by transfer to nitrocellulose membranes. Blots were blocked with nonfat dried milk prior to incubation with antibodies as indicated in the figures. Blots were developed using SuperSignal Chemiluminescent reagent (Pierce, Rockford, IL, USA) and quantified by densitometric analysis to show fold induction.

### Quantification of prostaglandin E2

Cells were transfected as indicated in the figures for 24 hours, and were cultured for an additional 24 hours in DMEM without FBS or antibiotics, and either with or without NS-398 (100 μM). Conditioned media or blood samples were used to quantify PGE2 using the PGE2 EIA kit according to the manufacturer's protocol (R&D systems).

### Immunoprecipitation

Cells were cultured in 10-cm dishes and were lysed in 800 μL lysis buffer (150 mM NaCl, 50 mM Tris/HCl (pH 7.4), 0.5% (v/v) Nonidet P-40, and protease inhibitor cocktail). Lysates were centrifuged at 14,000 ×g for 10 min, and 700 μL of each supernatant was incubated with 30 μL of protein A/G sepharose for 30 min. Antibodies (2 μg) were initially incubated with 50 μL of 50% (v/v) protein A/G-sepharose for 2 hours prior to addition to the lysate samples. Samples were incubated overnight, and the beads were extensively washed. Bound proteins were eluted from the beads and were resolved by SDS-PAGE.

### Mammalian two-hybrid assays

HepG2 cells were cultivated and transfected for 24 hours with 0.5 μg of pM and pVP16, 0.5 μg of reporter pG5-Luc, and 0.01 μg of control pRL-TK (Promega). Luciferase activity in each sample was determined using the dual-luciferase reporter assay system.

### Bisulfite modification and sequencing analysis

A total of 2 μg of genomic DNA was obtained from HepG2 and HepG2.2.1.5 cells, and was modified by sodium bisulfite. Primary and secondary PCR reactions were carried out in 50 μL reaction mixtures. Primers used to generate the primary PCR product were: COX-2-nest up 5'-GGAGGGATTAGATAGGAGAG-3' and COX-2-nest down 5'-AAATAAACTTTACTATCTAA-3'. To obtain products for sequencing, a secondary round of PCR was performed using the primary PCR products and the primers COX-2-up 5'-TTTAAATTGGGGTAGTTTTT-3' and COX-2-down 5'-CTACTAAAAAATTCCTAAA-3'. Secondary PCR products were gel-purified and cloned into a TA cloning vector (Promega). Individual plasmids were verified by DNA sequencing.

### Electrophoretic mobility shift assays (EMSA)

Nuclear extracts were prepared from HepG2 and HepG2.2.15 cells according to methods described previously [20]. C/EBP and NF-AT oligonucleotides were synthesized based on the human COX-2 promoter sequence:

Wild-type C/EBP: 5'-GCTTACGCAATTTTTTTAAGG-3';

Mutant C/EBP: 5'-GCGACTAGAATTTTTTTAAGG-3';

NF-AT: 5'-ACGAAAAGGCGGAAAGAAAC-3';

and Methyl NF-AT: 5'-ACGAAAAGGCGGAAAGAAAC-3'.

The complementary oligonucleotides were annealed and purified. Each probe was end-labeled with [γ^32^P]-ATP using T4 polynucleotide kinase (Takara Biotech). EMSA was performed by incubating 2 μg of nuclear extract with labeled probes (15,000 cpm; 10 fmol) in binding buffer (4 mM Tris-HCl, 12 mM HEPES-KOH, pH 7.9, 60 mM KCl, 12% glycerol, 0.5 mM EDTA, and 1 mM DTT) containing 1 μg of poly (dI-dC) for 25 min at room temperature. To ensure specific binding of transcription factors to the probes, the probes were chased by 100-fold molar excess of cold wild-type, mutant, or methylated oligonucleotides. Samples were electrophoresed on 5% nondenaturing polyacrylamide gels with running buffer (22.5 mM Tris borate and 0.5 mM EDTA), and the gels were dried and subjected to autoradiography.

### Chromatin immunoprecipitation (ChIP)

HepG2 and HepG2.2.1.5 cells were incubated for 24 hours after transfection (if transfection was necessary), after which they were serum-starved for an additional 24 hours. One percent formaldehyde was added to the culture medium, and the cells were washed twice with PBS, scraped, and lysed in buffer containing 1% SDS, 10 mM Tris-HCl pH 8.0, and protease inhibitor cocktail, for 10 min on ice. The lysates were sonicated on ice and the debris was removed by centrifugation at 12,000 ×g for 15 min at 4°C. One-fourth of the supernatant was used as DNA input control. The remaining sample was diluted 10-fold with dilution buffer (0.01% SDS, 1% Triton X-100, 1 mM EDTA, 10 mM Tris-HCl pH 8.0, and 150 mM NaCl) followed by incubation with antibodies against C/EBPβ, DNMT1, DNMT3A, or DNMT3B overnight at 4°C. Immunoprecipitated complexes were collected using protein A/G-sepharose. The pellets were washed for four times with dialysis buffer containing 2 mM EDTA and 50 mM Tris-HCl pH 8.0. After washing, the precipitates were incubated in an elution buffer (1% SDS and 0.1 M NaHCO_3_) at room temperature. Supernatants were transferred to clean tubes, and RNase A was added to destroy bound RNA in the sample. Samples were incubated at 65°C for 5 hours to reverse formaldehyde crosslinks, and DNA was precipitated with ethanol and extracted two times with phenol/chloroform. Pellets were suspended in TE buffer and subjected to PCR amplification using the COX-2 promoter detection primers C/EBP up: -155 TAAAAAACCCTGCCCCCACCGG -134, C/EBP down: -2 TCGCTAACCGAGAGAACCTTCCTT -25, DNMTs-b up: -177 CCGATTTTCTCATTTCCGTGGG -156, and DNMTs-b down: -56 CGAAATGACTGTTTCTTTCCGCC -78.

### DNMT activity assays

Nuclear extracts and total cellular proteins from HepG2 and HepG2.2.1.5 cells were prepared and protein concentrations were determined using the Bradford assay kit (Bio-Rad). Equal amounts of protein were used for DNMT activity measurement using the Epiquik DNA methyltransferase activity assay kit according to the manufacturer's protocol (Epigentek Group).

### Statistical analysis

All of the experiments were reproducible and were carried out in duplicates or quadruplicates. Each set of experiments was repeated at least three times with similar results. The results are presented at the means ± S.D. Student's t-test for paired samples was used to determine statistical significance. Differences were considered statistically significant at a value of P ≤ 0.05.

## List of abbreviations

HBV: hepatitis B virus; COX-2: cyclooxygenase-2; PGE2: prostaglandin E2; DNMT3B: DNA methyltransferase 3B; NF-AT: Nuclear Factor of Activated T-Cells; C/EBP: CCAAT/enhancer-binding protein.

## Competing interests

The authors declare that they have no competing interests.

## Authors' contributions

XY designed the study, carried out all the experiments, analyzed results and drafted the manuscript. FY and WL participated in the epigenetic modifications analysis of the study. YBY analyzed the data and helped to edit the manuscript. YXM and WS contributed to acquisition of data and interpretation of the results. DPX helped to provide clinical samples and data. YZ and JGW participated in the design of the study and the critical view of manuscript writing. All authors read and approved the final manuscript.

## References

[B1] Zuckerman AJ More than third of world's population has been infected with hepatitis B virus Bmj 1999 318 1213 10221961 10.1136/bmj.318.7192.1213a PMC1115601

[B2] Beasley RP Hwang LY Lin CC Chien CS Hepatocellular carcinoma and hepatitis B virus. A prospective study of 22 707 men in Taiwan Lancet 1981 2 1129 1133 10.1016/S0140-6736(81)90585-7 6118576

[B3] Robinson WS Molecular events in the pathogenesis of hepadnavirus-associated hepatocellular carcinoma Annu Rev Med 1994 45 297 323 10.1146/annurev.med.45.1.297 8198385

[B4] Gonzalez-Amaro R Garcia-Monzon C Garcia-Buey L Moreno-Otero R Alonso JL Yague E Pivel JP Lopez-Cabrera M Fernandez-Ruiz E Sanchez-Madrid F Induction of tumor necrosis factor alpha production by human hepatocytes in chronic viral hepatitis The Journal of experimental medicine 1994 179 841 848 10.1084/jem.179.3.841 7509363 PMC2191405

[B5] Fang JW Shen WW Meager A Lau JY Activation of the tumor necrosis factor-alpha system in the liver in chronic hepatitis B virus infection Am J Gastroenterol 1996 91 748 753 8677942

[B6] Lee Y Park US Choi I Yoon SK Park YM Lee YI Human interleukin 6 gene is activated by hepatitis B virus-X protein in human hepatoma cells Clin Cancer Res 1998 4 1711 1717 9676846

[B7] Majano PL Garcia-Monzon C Lopez-Cabrera M Lara-Pezzi E Fernandez-Ruiz E Garcia-Iglesias C Borque MJ Moreno-Otero R Inducible nitric oxide synthase expression in chronic viral hepatitis. Evidence for a virus-induced gene upregulation The Journal of clinical investigation 1998 101 1343 1352 10.1172/JCI774 9525976 PMC508711

[B8] Rahman MA Dhar DK Yamaguchi E Maruyama S Sato T Hayashi H Ono T Yamanoi A Kohno H Nagasue N Coexpression of inducible nitric oxide synthase and COX-2 in hepatocellular carcinoma and surrounding liver: possible involvement of COX-2 in the angiogenesis of hepatitis C virus-positive cases Clin Cancer Res 2001 7 1325 1332 11350902

[B9] Bae SH Jung ES Park YM Kim BS Kim BK Kim DG Ryu WS Expression of cyclooxygenase-2 (COX-2) in hepatocellular carcinoma and growth inhibition of hepatoma cell lines by a COX-2 inhibitor, NS-398 Clin Cancer Res 2001 7 1410 1418 11350912

[B10] Smith WL DeWitt DL Garavito RM Cyclooxygenases: structural, cellular, and molecular biology Annu Rev Biochem 2000 69 145 182 10.1146/annurev.biochem.69.1.145 10966456

[B11] Inoue H Yokoyama C Hara S Tone Y Tanabe T Transcriptional regulation of human prostaglandin-endoperoxide synthase-2 gene by lipopolysaccharide and phorbol ester in vascular endothelial cells. Involvement of both nuclear factor for interleukin-6 expression site and cAMP response element J Biol Chem 1995 270 24965 24971 10.1074/jbc.270.42.24965 7559624

[B12] Kim Y Fischer SM Transcriptional regulation of cyclooxygenase-2 in mouse skin carcinoma cells. Regulatory role of CCAAT/enhancer-binding proteins in the differential expression of cyclooxygenase-2 in normal and neoplastic tissues J Biol Chem 1998 273 27686 27694 10.1074/jbc.273.42.27686 9765305

[B13] Yamamoto K Arakawa T Ueda N Yamamoto S Transcriptional roles of nuclear factor kappa B and nuclear factor-interleukin-6 in the tumor necrosis factor alpha-dependent induction of cyclooxygenase-2 in MC3T3-E1 cells J Biol Chem 1995 270 31315 31320 10.1074/jbc.270.52.31315 8537402

[B14] Murono S Inoue H Tanabe T Joab I Yoshizaki T Furukawa M Pagano JS Induction of cyclooxygenase-2 by Epstein-Barr virus latent membrane protein 1 is involved in vascular endothelial growth factor production in nasopharyngeal carcinoma cells Proc Natl Acad Sci USA 2001 98 6905 6910 10.1073/pnas.121016998 11381123 PMC34451

[B15] Bagetta G Corasaniti MT Paoletti AM Berliocchi L Nistico R Giammarioli AM Malorni W Finazzi-Agro A HIV-1 gp120-induced apoptosis in the rat neocortex involves enhanced expression of cyclo-oxygenase type 2 (COX-2) Biochem Biophys Res Commun 1998 244 819 824 10.1006/bbrc.1998.8321 9535750

[B16] Flora G Pu H Hennig B Toborek M Cyclooxygenase-2 is involved in HIV-1 Tat-induced inflammatory responses in the brain Neuromolecular Med 2006 8 337 352 10.1385/NMM:8:3:337 16775385

[B17] Nunez O Fernandez-Martinez A Majano PL Apolinario A Gomez-Gonzalo M Benedicto I Lopez-Cabrera M Bosca L Clemente G Garcia-Monzon C Martin-Sanz P Increased intrahepatic cyclooxygenase 2, matrix metalloproteinase 2, and matrix metalloproteinase 9 expression is associated with progressive liver disease in chronic hepatitis C virus infection: role of viral core and NS5A proteins Gut 2004 53 1665 1672 10.1136/gut.2003.038364 15479690 PMC1774290

[B18] Lu L Wei L Peng G Mu Y Wu K Kang L Yan X Zhu Y Wu J NS3 protein of hepatitis C virus regulates cyclooxygenase-2 expression through multiple signaling pathways Virology 2008 371 61 70 10.1016/j.virol.2007.09.025 17964630 PMC7103338

[B19] Yan X Hao Q Mu Y Timani KA Ye L Zhu Y Wu J Nucleocapsid protein of SARS-CoV activates the expression of cyclooxygenase-2 by binding directly to regulatory elements for nuclear factor-kappa B and CCAAT/enhancer binding protein Int J Biochem Cell Biol 2006 38 1417 1428 10.1016/j.biocel.2006.02.003 16546436 PMC7108415

[B20] Liu M Yang Y Gu C Yue Y Wu KK Wu J Zhu Y Spike protein of SARS-CoV stimulates cyclooxygenase-2 expression via both calcium-dependent and calcium-independent protein kinase C pathways Faseb J 2007 21 1586 1596 10.1096/fj.06-6589com 17267381

[B21] Cheng AS Chan HL Leung NW Liew CT To KF Lai PB Sung JJ Expression of cyclooxygenase-2 in chronic hepatitis B and the effects of anti-viral therapy Aliment Pharmacol Ther 2002 16 251 260 10.1046/j.1365-2036.2002.01163.x 11860408

[B22] Diao J Garces R Richardson CD X protein of hepatitis B virus modulates cytokine and growth factor related signal transduction pathways during the course of viral infections and hepatocarcinogenesis Cytokine Growth Factor Rev 2001 12 189 205 10.1016/S1359-6101(00)00034-4 11325602

[B23] Lara-Pezzi E Gomez-Gaviro MV Galvez BG Mira E Iniguez MA Fresno M Martinez AC Arroyo AG Lopez-Cabrera M The hepatitis B virus X protein promotes tumor cell invasion by inducing membrane-type matrix metalloproteinase-1 and cyclooxygenase-2 expression The Journal of clinical investigation 2002 110 1831 1838 12488433 10.1172/JCI200215887 PMC151651

[B24] Hsieh CL Dependence of transcriptional repression on CpG methylation density Mol Cell Biol 1994 14 5487 5494 7518564 10.1128/mcb.14.8.5487-5494.1994 PMC359068

[B25] Wenger RH Kvietikova I Rolfs A Camenisch G Gassmann M Oxygen-regulated erythropoietin gene expression is dependent on a CpG methylation-free hypoxia-inducible factor-1 DNA-binding site Eur J Biochem 1998 253 771 777 10.1046/j.1432-1327.1998.2530771.x 9654078

[B26] Cheng AS Chan HL Leung WK To KF Go MY Chan JY Liew CT Sung JJ Expression of HBx and COX-2 in chronic hepatitis B, cirrhosis and hepatocellular carcinoma: implication of HBx in upregulation of COX-2 Mod Pathol 2004 17 1169 1179 10.1038/modpathol.3800196 15218507

[B27] Zha S Yegnasubramanian V Nelson WG Isaacs WB De Marzo AM Cyclooxygenases in cancer: progress and perspective Cancer Lett 2004 215 1 20 10.1016/j.canlet.2004.06.014 15374627

[B28] Ramji DP Foka P CCAAT/enhancer-binding proteins: structure, function and regulation Biochem J 2002 365 561 575 12006103 10.1042/BJ20020508 PMC1222736

[B29] Lekstrom-Himes J Xanthopoulos KG Biological role of the CCAAT/enhancer-binding protein family of transcription factors J Biol Chem 1998 273 28545 28548 10.1074/jbc.273.44.28545 9786841

[B30] Descombes P Schibler U A liver-enriched transcriptional activator protein, LAP, and a transcriptional inhibitory protein, LIP, are translated from the same mRNA Cell 1991 67 569 579 10.1016/0092-8674(91)90531-3 1934061

[B31] Barnabas S Hai T Andrisani OM The hepatitis B virus X protein enhances the DNA binding potential and transcription efficacy of bZip transcription factors J Biol Chem 1997 272 20684 20690 10.1074/jbc.272.33.20684 9252388

[B32] Su PF Lee TC Lin PJ Lee PH Jeng YM Chen CH Liang JD Chiou LL Huang GT Lee HS Differential DNA methylation associated with hepatitis B virus infection in hepatocellular carcinoma Int J Cancer 2007 121 1257 1264 10.1002/ijc.22849 17534893

[B33] Creusot F Acs G Christman JK Inhibition of DNA methyltransferase and induction of Friend erythroleukemia cell differentiation by 5-azacytidine and 5-aza-2'-deoxycytidine The Journal of biological chemistry 1982 257 2041 2048 6173384

[B34] Michalowsky LA Jones PA Differential nuclear protein binding to 5-azacytosine-containing DNA as a potential mechanism for 5-aza-2'-deoxycytidine resistance Molecular and cellular biology 1987 7 3076 3083 2444874 10.1128/mcb.7.9.3076-3083.1987 PMC367939

[B35] Frommer M McDonald LE Millar DS Collis CM Watt F Grigg GW Molloy PL Paul CL A genomic sequencing protocol that yields a positive display of 5-methylcytosine residues in individual DNA strands Proceedings of the National Academy of Sciences of the United States of America 1992 89 1827 1831 10.1073/pnas.89.5.1827 1542678 PMC48546

[B36] Li E Chromatin modification and epigenetic reprogramming in mammalian development Nat Rev Genet 2002 3 662 673 10.1038/nrg887 12209141

[B37] Robertson KD Uzvolgyi E Liang G Talmadge C Sumegi J Gonzales FA Jones PA The human DNA methyltransferases (DNMTs) 1, 3a and 3b: coordinate mRNA expression in normal tissues and overexpression in tumors Nucleic Acids Res 1999 27 2291 2298 10.1093/nar/27.11.2291 10325416 PMC148793

[B38] Park IY Sohn BH Yu E Suh DJ Chung YH Lee JH Surzycki SJ Lee YI Aberrant epigenetic modifications in hepatocarcinogenesis induced by hepatitis B virus X protein Gastroenterology 2007 132 1476 1494 10.1053/j.gastro.2007.01.034 17408664

[B39] Bouchard MJ Schneider RJ The enigmatic X gene of hepatitis B virus J Virol 2004 78 12725 12734 10.1128/JVI.78.23.12725-12734.2004 15542625 PMC524990

[B40] Andrisani OM Barnabas S The transcriptional function of the hepatitis B virus X protein and its role in hepatocarcinogenesis (Review) Int J Oncol 1999 15 373 379 10402250 10.3892/ijo.15.2.373

[B41] Maguire HF Hoeffler JP Siddiqui A HBV X protein alters the DNA binding specificity of CREB and ATF-2 by protein-protein interactions Science 1991 252 842 844 10.1126/science.1827531 1827531

[B42] Ehrlich M Expression of various genes is controlled by DNA methylation during mammalian development J Cell Biochem 2003 88 899 910 10.1002/jcb.10464 12616529

[B43] Akhtar M Cheng Y Magno RM Ashktorab H Smoot DT Meltzer SJ Wilson KT Promoter methylation regulates Helicobacter pylori-stimulated cyclooxygenase-2 expression in gastric epithelial cells Cancer Res 2001 61 2399 2403 11289104

[B44] Song SH Jong HS Choi HH Inoue H Tanabe T Kim NK Bang YJ Transcriptional silencing of Cyclooxygenase-2 by hyper-methylation of the 5' CpG island in human gastric carcinoma cells Cancer Res 2001 61 4628 4635 11389100

[B45] Toyota M Shen L Ohe-Toyota M Hamilton SR Sinicrope FA Issa JP Aberrant methylation of the Cyclooxygenase 2 CpG island in colorectal tumors Cancer Res 2000 60 4044 4048 10945606

[B46] Rouleau J MacLeod AR Szyf M Regulation of the DNA methyltransferase by the Ras-AP-1 signaling pathway J Biol Chem 1995 270 1595 1601 10.1074/jbc.270.4.1595 7829490

[B47] Zheng DL Zhang L Cheng N Xu X Deng Q Teng XM Wang KS Zhang X Huang J Han ZG Epigenetic modification induced by hepatitis B virus X protein via interaction with de novo DNA methyltransferase DNMT3A J Hepatol 2009 50 377 387 10.1016/j.jhep.2008.10.019 19070387

[B48] Lim W Kwon SH Cho H Kim S Lee S Ryu WS Cho H HBx targeting to mitochondria and ROS generation are necessary but insufficient for HBV-induced cyclooxygenase-2 expression Journal of molecular medicine (Berlin, Germany) 88 359 369 19940973 10.1007/s00109-009-0563-z

[B49] Keasler VV Hodgson AJ Madden CR Slagle BL Enhancement of hepatitis B virus replication by the regulatory X protein in vitro and in vivo Journal of virology 2007 81 2656 2662 10.1128/JVI.02020-06 17182675 PMC1865975

[B50] Zhu Y Saunders MA Yeh H Deng WG Wu KK Dynamic regulation of cyclooxygenase-2 promoter activity by isoforms of CCAAT/enhancer-binding proteins J Biol Chem 2002 277 6923 6928 10.1074/jbc.M108075200 11741938

[B51] Rahnama F Shafiei F Gluckman PD Mitchell MD Lobie PE Epigenetic regulation of human trophoblastic cell migration and invasion Endocrinology 2006 147 5275 5283 10.1210/en.2006-0288 16887905

